# Context-sensitive adjustment of pointing in great apes

**DOI:** 10.1038/s41598-019-56183-7

**Published:** 2020-01-23

**Authors:** Tibor Tauzin, Manuel Bohn, György Gergely, Josep Call

**Affiliations:** 1 Central European University, Budapest, Hungary; 2 Max Planck Institute for Evolutionary Anthropology, Leipzig, Germany; 3 Stanford University, Stanford, CA, USA; 4 Leipzig University, Leipzig, Germany; 5 University of St. Andrews, St. Andrews, UK

**Keywords:** Human behaviour, Animal behaviour

## Abstract

Great apes are able to request objects from humans by pointing. It is unclear, however, whether this is an associated response to a certain set of cues (e.g. the presence and attention of a human addressee) or a communicative signal which can be adjusted to relevant aspects of the spatial and social context. In three experiments, we tested captive great apes’ flexible use of pointing gestures. We manipulated the communicative context so that the default pointing response of apes would have indicated an undesired object, either due to 1) the spatial arrangements of the target objects, 2) the perspective of the addressee or 3) the knowledge of the addressee about the target objects’ location. The results of the three experiments indicate that great apes can successfully adjust their pointing to the spatial configuration of the referent environment such as distance and location of food. However, we found no evidence that they take the perspective or the knowledge of the addressee into account when doing so. This implies that pointing in great apes is a context-sensitive, but maybe less versatile, communicative signal compared to human pointing.

## Introduction

Human communication rests on the ability to express and infer informative intentions manifested by context-sensitive verbal or non-verbal signals^1–3^. The role and relevance of intentionality in animal communication, however, has been highly debated in the past decades e.g.^4–7^. One reason for this debate is that it is not always clear what a good behavioral indicator of intentional communication is. Inspired by seminal work with infants^8,9^, Townsend and colleagues^7^ recently proposed three operational criteria for intentional communication: 1) cessation or elaboration of signaling depending on goal-achievement, 2) recipient-directedness, and 3) reliable and congruent relation between the communicative intention of the signaler and response. Great ape gestural communication meets the criteria of intentional gesture use^8,9^ as apes elaborate or persist their signaling if the intended outcome is not achieved^10,11^, they adjust their gestures to the attentional state of the addressee^12–15^, alternate gaze between the addressee and the referent^16^ and use certain gestures to achieve certain goals^17^. Here we study a further potential indicator of intentional communication, namely, flexible adjustment of gestures to relevant variations in the spatial and social context.

In human communication the meaning of a signal can depend on the spatial and social context it is used in^18,19^. To avoid ambiguity, communicators need to consider contextual constraints when they produce signals. For example, humans’ pointing gesture can be modified spontaneously to refer to a lateral or blocked object by adjusting the position of the index finger to be in line with the indicated object. Consequently, it can be assumed that in addition to the criteria listed above, flexible adjustment of signals depending on contextual constraints would be a good indicator that the signal itself is produced intentionally.

Previous empirical findings suggest that the production of gestures in captive great apes is indeed context-sensitive^20^. For example, the same gestures are used to achieve different goals in different situations^17,21^. However, there is no direct evidence so far that great apes spontaneously adjust their signal use to the relevant aspects of the physical and social context to disambiguate a certain referent for the addressee. In previous studies^10,11^, great apes modified their communicative behavior by changing from one set of signals to another (e.g. from hand gestures to cage banging). However, this may reflect persistence of signaling until a desired goal-state is achieved, but not necessarily an understanding of the context-dependent meaning of communicative signals.

A promising type of action to study context-sensitive adjustment of communicative signals in great apes, is the pointing gesture^22^. Although it is rarely produced in the wild^23^, captive animals regularly use this gesture e.g.^24–26^ mostly to request an object from a human (in contrast to human infants, see^27,28^) but not from a conspecific^29,30^ but see^31^. Accordingly, great apes produce pointing in a restricted social context, that is, when a human addressee is present^24,25^.

This suggests context-sensitive intentional use of pointing in captive great apes. One could object, however, that pointing in apes does not serve an indexical function as it may be a consequence of an unsuccessful direct reach in a situation where the referent object is blocked by an obstacle (typically by a mesh panel, see^29^). Since this “failed reach” is rewarded with a desired object (usually a food item) by a human, great apes may associatively learn, how (by reaching through the mesh) and when (in the presence of a human) to perform such a manual behavior. This non-communicative account is supported by results of Liszkowski and colleagues^32^ who found that apes do not point for absent entities (presumably because there are no visual cues to induce this action), and by studies showing that apes try to get as close to the target object as possible before pointing^33,34^.

However, another set of findings calls the associative account into question. Leavens *et al*.^35^ showed that great apes can use pointing from a distance, suggesting that this gesture is not simply an action to substitute for reaching, but used for purposes of interspecies communication (see also^36^). Another set of results suggest that great apes do point for absent entities^37–39^, implying that pointing is not exclusively driven by perceptual cues. These findings, however, do not directly show that pointing is used intentionally to disambiguate the intended referent for the addressee in a given context.

Two previous studies looked at fine-grained adjustment of pointing as a function of spatial context and found that subjects changed the height of their pointing hand depending on the distance of the referent^36,40^. However, because there was only one target object presented, these experiments were insufficient to show that the modification of pointing served a communicative purpose and was used to disambiguate the referent for the addressee. To create an incentive for disambiguation, failing to clearly refer to the desired object should lead to a less desired outcome. Consequently, to examine how apes would adjust their pointing gesture to a given context in order to unambiguously indicate a desired object, we conjectured that it is necessary to have more than one potential target-objects present.

**Figure 1:**
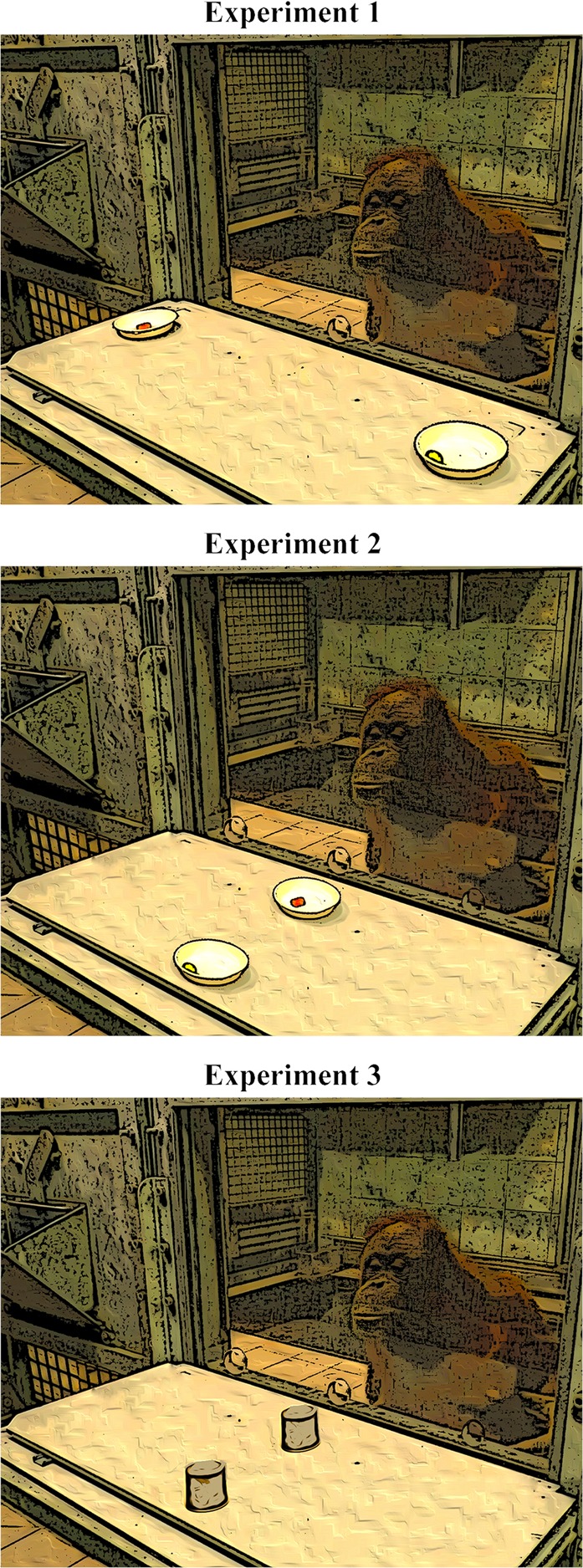
The setup and arrangement of the three experiments with the hole in the middle in Experiment 1 and with the 3-holed Plexiglas panel in Experiment 2 and 3.

We conducted three experiments in which we tested great apes’ ability to modify their default pointing behavior used for food requests in order to indicate a high quality (HQ) instead of a low quality (LQ) food item. Food items were either (1) closely behind a transparent barrier with one pointing hole only, (2) aligned in a row to induce modified pointing when HQ food was behind the LQ food item or (3) aligned in a row and only accessible by requesting help from an addressee who was ignorant about the location of food the items (see also Fig. 1). We assumed that if apes use pointing to indicate a particular object to an addressee, they should modify their pointing gesture according to the spatial layout of alternative referent locations in a way that help the addressee to disambiguate and identify the intended food item.

## Experiment 1

In Experiment 1 our aim was to test whether great apes can adjust their pointing gesture to the spatial properties of the context in order to indicate a desirable food item which was further away from a pointing hole. We presented high quality food (grape) and low quality food item (carrot) on either of two sides of a table behind a Plexiglas panel, which had one hole only (on the left or right side or in the middle). We hypothesized that apes would modify their pointing behavior if the HQ food was not directly positioned behind the pointing hole, for instance, by bending their pointing finger to be aligned with the HQ food item or by pointing from behind the Plexiglass panel at the HQ food item. In contrast, apes were hypothesized not to modify their pointing gestures if pointing simply substitutes for reaching. We also investigated the effect of hole placement in the Plexiglas panel to see whether the variation of the spatial context can have an effect on apes’ pointing.

### Methods

#### Participants

Twenty-two great apes participated in Experiment 1. We tested 7 bonobos (*Pan paniscus*, mean age = 19.29 years, *SD* = 8.98, 2 males), 6 chimpanzees (*Pan troglodytes*, mean age = 30.83 years, *SD* = 14.01, 1 male), 3 gorillas (*Gorilla gorilla*, mean age = 14.67 years, *SD* = 2.89, 1 male) and 6 orangutans (*Pongo abelii*, mean age = 22.67 years, *SD* = 10.69, 2 males). Apes were housed at the Wolfgang Köhler Primate Research Center, Zoo Leipzig, Germany. All participants had prior experience with selecting objects or food items by pointing.

#### Setup

The paradigm we used was based on previous studies e.g.^29,30^. We installed a Plexiglas panel (69 × 48 cm) in the sleeping rooms of each species with a single hole (diameter = 4 cm) in the bottom part of the panel. Depending on session, the hole was either on the left, the right or in the middle of the panel. The experimenter sat on the other side of the Plexiglas panel in front of the ape. Food items were placed on two small white plates (diameter = 10 cm) that were mounted on a sliding table. The two plates were on the left and right sides of the sliding table equidistant from the midline and aligned with the respective lateral holes on the Plexiglas panel. The plates were out-of-reach from the apes, therefore they could not touch them with the finger. The food items in all trials were a slice of carrot (low quality food item – LQ) and a piece of grape (high quality food item – HQ; see also Fig. 1). A large opaque plastic occluder was used to cover the sliding table during baiting between trials.

#### Procedure

Each subject participated in three sessions, one with each of the Plexiglas panels. All sessions consisted of 12 test trials where the subject’s task was to indicate one of two food items by pointing. In all test trials the experimenter first placed the HQ and LQ food items on the lateral plates while he held an opaque occluder blocking the subjects’ view of placement of the target objects. After the food items were located, the experimenter removed the occluder. If the ape pointed at one of the two pieces of food, the experimenter immediately gave the requested food to the subject through a feeding hole which was approximately 0.5 meter away from the nearest edge of the test Plexiglas panel. If the ape did not perform any food-oriented actions at all or if the food oriented action was not pointing (banging the Plexiglas panel etc.) the experimenter waited 30 seconds. When the 30 seconds expired the Experimenter gave the HQ food item to the ape to maintain the motivation of the subject. This occurred in 16.79% of the trials.

The location of the two food items was pseudorandomized and counterbalanced across trials in a given session, therefore both type of food items appeared in each side equally often, however, none of them was presented more than two times in a row at the same location.

#### Data analysis

All trials were video recorded and analyzed offline. We defined pointing as a hand action with a protruded or bent finger aimed at the direction of a given food item. The main measure of interest was modified pointing, which in this setup could be 1) *bent pointing* when the subject inserted the finger into the pointing hole and bent it, therefore, the fingertip pointed towards the distant plate located on either the left or the right of the hole. Or, it could be a 2) *pointing from behind the Plexiglas panel* when the subject did not insert its finger into the pointing hole, but moved the finger close to the desired food item and pointed at it “through” the Plexiglas. We only coded subject’s first response because the experimenter gave the indicated food item to the subject immediately after the pointing, making gesture repetition unlikely. Furthermore, we wanted to avoid shaping the use of modified points. Unmodified points were not included in later analysis.

We calculated the sum of modified points to the HQ food item by a given individual. As a baseline, we also calculated the sum of modified points at the LQ food item. The maximum number of modified pointing at HQ and LQ was 24, as modified pointing could occur 12 times in the hole in the middle session and 6 times in each session with the hole on the left and right. We analyzed the data using ordinal generalized estimating equation (GEE) tests^41^ with main effects of Food location (HQ vs. LQ food item closer to the pointing hole) and Panel (pointing hole in the middle, left or right side of the panel). We investigated these effects in bent pointing and pointing from behind the Plexiglas panel separately. A second coder analyzed 25% of all trials. There was high agreement (97.47%) between the two coders (*κ* = 0.924, *N* = 198, *p* < 0.001).

### Results

**Figure 2:**
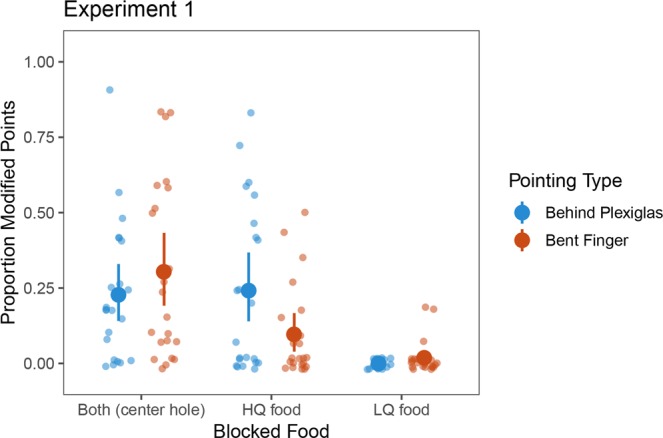
Proportion of modified points when HQ, LQ or both food items were blocked in Experiment 1. Larger dots indicate the means, error bars represent 95% CIs.

According to a GEE analysis there was a significant main effect of Food location (*χ*^2^_*(1)*_ = 36.697*, p* < 0.001), a significant main effect of Panel (*χ*^2^_*(2)*_ = 7.501*, p* = 0.024, for further details see the Supporting Information) and a non-significant Food location × Panel interaction (*χ*^2^_*(2)*_ = 3.468*, p* = 0.177) when both type of modified pointing were analyzed together. The average number of trials with modified points at the HQ food (when it was not in front of a pointing hole) was 9.41 (*SD* = 5.85), that is, it occurred in 39.21% of the relevant trials. In contrast, the average number of trials with modified points directed at the LQ food (when it was not in front of a pointing hole) was 1.18 (*SD* = 1.71), thus it was produced in 4.92% of the trials (Fig. 2).

### Discussion

The results of Experiment 1 showed that great apes can modify their pointing gestures performing bent pointing and pointing from behind a transparent panel when they tried to indicate a desired food item. Apes produced modified pointing spontaneously and in a flexible manner to indicate the HQ in contrast to LQ food item. This is especially notable, because in previous studies the same apes were asked to point through the hole that was closest to the desired object (see e.g.^37,38,42,43^).

The present findings suggest that pointing in great apes is not simply an associative response to certain perceptual cues, but a versatile tool that can be adapted to the aspects of the spatial context for communicative purposes. This is also indicated by the finding that great apes not simply substituted their typical proximal pointing with a different gesture, but modified the existing one by pointing from behind the Plexiglas panel or bending the finger when using the pointing hole. In line with the communicative account of pointing, this suggests that great apes can intentionally change their pointing gestures to inform the addressee about a desired referent object while taking the spatial-referential context into account.

## Experiment 2

The aim of Experiment 2 was to investigate whether great apes can take the perspective of the communicative partner into account in a spatial context where their typical pointing can be ambiguous, potentially referring to a less desired object. We designed a setup in which HQ and LQ food items were placed on two plates arranged in one row clearly visible to the ape and the experimenter but from a different angle. In the HQ front condition, the HQ item was closer to the ape so that central pointing would refer to the HQ food item. In the LQ front condition, the LQ food was closer to the ape so that lateral pointing (from the sides or above) was needed to indicate the HQ food in a context-sensitive manner. In the No food front condition, only the HQ food item was placed on the back plate while no food was placed on the front plate. Therefore, although the desirable food item was further away, central pointing would be sufficient to indicate the HQ food item unambiguously to the addressee.

We hypothesized that if apes are sensitive to the perspective of the addressee, they will produce more lateral points in the LQ front condition compared to the HQ front and to the No food front conditions to unambiguously indicate the desired HQ food item. However, if apes take only the spatial context into account, and have a limited understanding of the addressee’s perspective, they should produce more lateral points when the HQ food item is further away irrespective of the presence and position of the LQ food item. Consequently, a difference was expected both between the LQ and HQ front conditions and between the HQ and No food front conditions, if the function of lateral points was to indicate an object that is further away.

### Methods

#### Participants

In total, 21 great apes (20 of them participated in Experiment 1) from the same institution were tested in Experiment 2, including 7 bonobos (*Pan paniscus*, mean age = 19.29 years, *SD* = 8.98, 2 males), 5 chimpanzees (*Pan troglodytes*, mean age = 31.4 years, *SD* = 15.04, 1 male), 3 gorillas (*Gorilla gorilla*, mean age = 14.67 years, *SD* = 2.89, 1 male) and 6 orangutans (*Pongo abelii*, mean age = 22.67 years, *SD* = 10.69, 2 males).

#### Setup

The setup was similar to that of Experiment 1 except that in a session we used either a Plexiglas panel with 3 holes evenly spaced at the bottom, an intact Plexiglas panel without holes or a mesh panel. The plastic plates containing the food items were located behind each other in the midline of the sliding table, therefore one of them was further away from the subject while the other was closer to it. The distance between the plates was approximately 25 cm (see also Fig. 1).

#### Procedure

The procedure of Experiment 2 was similar to the procedure of Experiment 1 except that 3 different test trials were presented in each session (HQ front, LQ front, No food front). The counterbalance and randomization of the test trials were the same as in Experiment 1.

#### Data analysis

**Figure 3:**
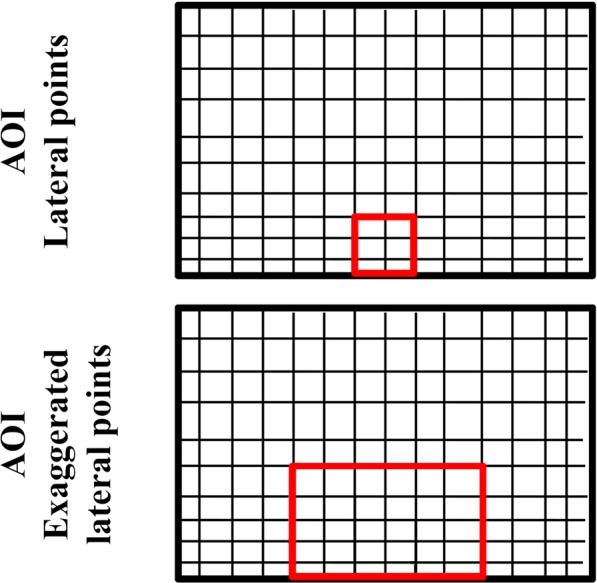
The boundaries of the central AOI (marked with red) and the AOI of lateral and exaggerated lateral points (around the central AOI) in the trials with the mesh panel.

In Experiment 2 we differentiated between central and lateral pointing. Central pointing was defined as pointing through the central AOI (Fig. 3) of the mesh, pointing from behind a similar sized AOI of the intact Plexiglas panel or using the middle pointing hole in the sessions with the 3-holed Plexiglas. Lateral points included all pointing gestures from outside of these predefined areas. We also examined and coded a subset of lateral points, namely exaggerated lateral points in the trials with the mesh panel. These gestures were highly inefficient for reaching due to their motion trajectory, as the ape had to move its hand away from the desired food item to unambiguously indicate an object (see also Fig. 3).

We calculated the number of lateral points for each subject (which could range between 0 and 12 per condition). This data was analyzed later by ordinal GEE testing the effect of Food location (HQ, LQ, No food front) and Panel (mesh, intact Plexiglas, Plexiglas with 3 holes). In addition, we tested the effect of Food location on exaggerated points. A GEE analysis could not be computed here due to the low number of exaggerated points when the HQ food was in front. Therefore, we used a Friedman test to test for differences between conditions and Wilcoxon sign-rank tests to directly compare conditions. A second coder coded 25% of all the trials. There was a very high agreement (98.94%) between the two coders (*κ* = 0.959, *N* = 189, *p* < 0.001).

### Results

**Figure 4:**
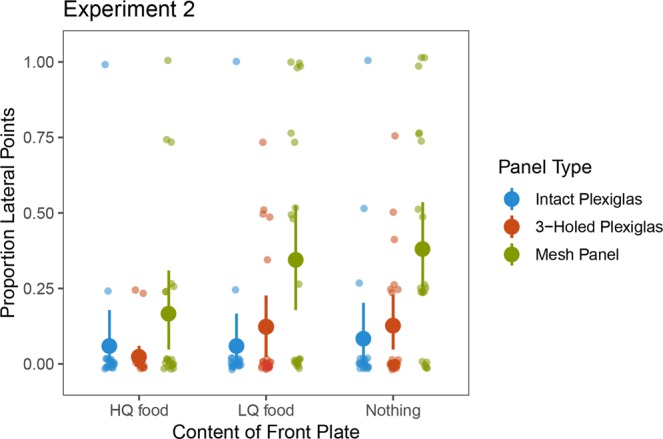
Proportion of lateral points in the three conditions of Experiment 2, when using the different panels. Larger dots indicate the means, error bars represent 95% CIs.

A GEE analysis of the data from all the panels revealed a significant main effect of Food location (*χ*^2^_*(2)*_ = 18.196, *p* < 0.001) and Panel (*χ*^2^_*(2)*_ = 13.545, *p* = 0.001) and a significant interaction of Food location × Panel (*χ*^2^_*(4)*_ = 9.664, *p* = 0.046; see also Supporting Information). There was a significant difference between the LQ front [*M* = 2.14 (17.85%), *SD* = 2.5] and HQ front [*M* = 1.1 (9.13%), *SD* = 1.7] conditions in the number of lateral points (*χ*^2^_*(1)*_ = 4.291*, p* = 0.038). Also, there was a significant difference (*χ*^2^_*(1)*_ = 11.634, *p* = 0.001) between the number of lateral points in the HQ front and in the No food front condition [*M* = 2.43 (20.24%), *SD* = 2.4]. The difference between the LQ front and No food front condition was not significant (*χ*^2^_*(1)*_ = 2.667*, p* = 0.102; see also Fig. 4).

When the mesh panel was used we also measured the number of exaggerated lateral points, which would have been highly inefficient as reaching actions, but could indicate the food item further away unambiguously. Food location had a significant effect on the occurrence of such points according to a Friedman test (*χ*^2^_(2)_ = 11.231, *p* = 0.002). The use of exaggerated points was significantly higher (*Z* = 2.388, *p* = 0.016) in the LQ front (*M* = 0.76 (19.05%), *SD* = 1.34) than in the HQ front condition (*M* = 0.0 (0.0%), *SD* = 0.0). There was also a significant difference (*Z* = 2.214, *p* = 0.031) between HQ front and No food front conditions (*M* = 0.71 (17.86%), *SD* = 1.27). No significant difference was found between the LQ front and No food front conditions.

### Discussion

The significant main effect of Food location in Experiment 2 supported the main finding of Experiment 1 suggesting that apes are sensitive to the spatial-referential context when pointing and can modify their pointing gesture when a central pointing would have indicated an undesired LQ food item. However, there was no significant difference in the number of lateral points between the LQ front and No food front conditions.

The results obtained are in line with the second, alternative hypothesis indicating that apes might have only a limited understanding of the addressee’s perspective when producing communicative gestures in the present setup (while they might also track the presence of an addressee, see^24,25^). This suggests that they can modify their pointing action as a function of the spatial layout, although other variations in signaling such as pointing more slowly or more frequently for objects further away could also be used^44^. Importantly, apes might be limited in their ability to infer how pointing should be modified to be optimally informative for an addressee with a different perspective. Thus, great apes seem unable or less inclined to integrate the information about the spatial with the social-perspectival context, at least when pointing in the present experiment.

In light of previous studies looking at pointing for absent referents^37–39^, it is also possible that apes expected to receive the food that was previously on the plate. Therefore, to avoid this unwanted outcome, they might produce more lateral points in that condition which then resulted in the non-significant difference between the LQ front and No food front conditions. Importantly, this latter interpretation also implies that apes can take the spatial layout of the setup into account and this information modulated their pointing behavior.

One might argue, however, that apes’ performance would have been the same if they simply tried to reach for the HQ food item instead of pointing. Since lateral points in Experiment 2 could be also explained as pointing through the nearest possible hole next to the central AOI it could be argued that instead of communicative pointing these gestures were failed reaching actions that were performed by using the most efficient motion trajectory available. This account, however, could not accommodate the findings about the exaggerated lateral points, which were also modulated by the location of the HQ food item, but were highly inefficient to perform as an attempt to reach for the target showing that, instead of being failed reaching attempts, the spatial context indeed induced modified pointing. Importantly, the number of lateral points was relatively low throughout Experiment 2, which suggests that even though apes are able to flexibly adjust their pointing to contextual variations, this behavior is not the default.

## Experiment 3

In experiment 2 we did not find evidence in great apes for taking the addressee’s perspective into consideration, therefore in Experiment 3, we investigated this question further using a different manipulation. We assumed that apes might show a higher rate of lateral pointing if there is an increased need to disambiguate their request. Such an increased need would be if the experimenter did not know about the hiding location of the desired food item. Consequently, we designed an experimental setup in which food items were hidden under two opaque cups in a row and manipulated the experimenter’s knowledge about the location of food by changing experimenters in half of the trials. In the latter type of trials, Experimenter 1 hid the food items while Experimenter 2 was the addressee of the ape’s pointing. In the other half of the test trials, there was no change in the experimenter. This resulted in four different conditions (LQ front - HQ back or HQ front - LQ back with or without change in the experimenter) with an additional factor of the Panel. Given the low rate of pointing with the intact Plexiglas panel, we did not use it in experiment 3. We hypothesized that the interaction of Food location × Experimenter will be significant if apes can disambiguate their pointing for an ignorant addressee, however, a main effect of Food location will be found if only the spatial configuration affects apes’ pointing in this setup.

### Methods

#### Participants

In Experiment 3 we tested 19 great apes in total (15 tested in Experiment 1). This included 7 bonobos (*Pan paniscus*, mean age = 19.28 years, *SD* = 8.98, 2 males), 5 chimpanzees (*Pan* troglodytes, mean age = 31.4 years, *SD* = 15.04, 1 male) and 7 orangutans (*Pongo abelii*, mean age = 16.43 years, *SD* = 9.34, 2 males).

#### Setup

The sessions in Experiment 3 were similar to those of Experiment 2, except that only the mesh panel and the Plexiglass panel with the 3 evenly spaced holes were used. We did not use the intact Plexiglas panel, since it did not differentiate among the conditions in Experiment 2. Experiment 3 was conducted by two experimenters: Experimenter 1 (female) and Experimenter 2 (male). The familiarity of the two experimenters varied across participants (for further details see the supplementary information). Food items were placed under small, grey, opaque cups (diameter = 5 centimeter) instead of a plate to prevent the addressee to know where the different food items were hidden (Fig. 1).

#### Procedure

In Experiment 3 the subjects were tested in 4 sessions (two sessions with each panel) each of which consisted of 12 consecutive test trials. A trial started with one of two experimenters – for example Experimenter 1 – entering the testing room sitting down in front of the ape and hiding the HQ and LQ food items (in full view of the ape) under the two opaque cups which were located behind each other in the midline of the sliding table. Then, the hider left the testing room and following a short (approximately 3–5 second long) delay while no experimenter was present, either experimenter 1 or experimenter 2 entered into the room and sat down in front of the ape waiting for the ape to choose between the food items by pointing at one of the cups. Therefore, the ape could be presented with 4 different trials involving the same experimenter or different experimenters and the HQ food under the front cup or the rear cup while the LQ item hidden under the other cup. The location of the food items, the trials with the same and different experimenters as well as the role (hider or addressee) of experimenter 1 and 2 in the same experimenter and different experimenter trials was counterbalanced within each session. In all other aspects Experiment 3 was similar to Experiment 2.

#### Data analysis

Data analysis was the same as in Experiment 2, except that we performed a Food location (HQ front or back) × Experimenter (same or different) × Panel (mesh or intact Plexiglas) GEE. Due to the low number of exaggerated points in the trials with the mesh panel, the difference between conditions could not be computed. A second coder coded 25% of all the trials. There was a very high agreement (99.06%) between the two coders (*κ* = 0.955, *N* = 237, *p* < 0.001). To test the effect of Experimenter familiarity we also employed a GLMM model (see supplementary information).

### Results

**Figure 5:**
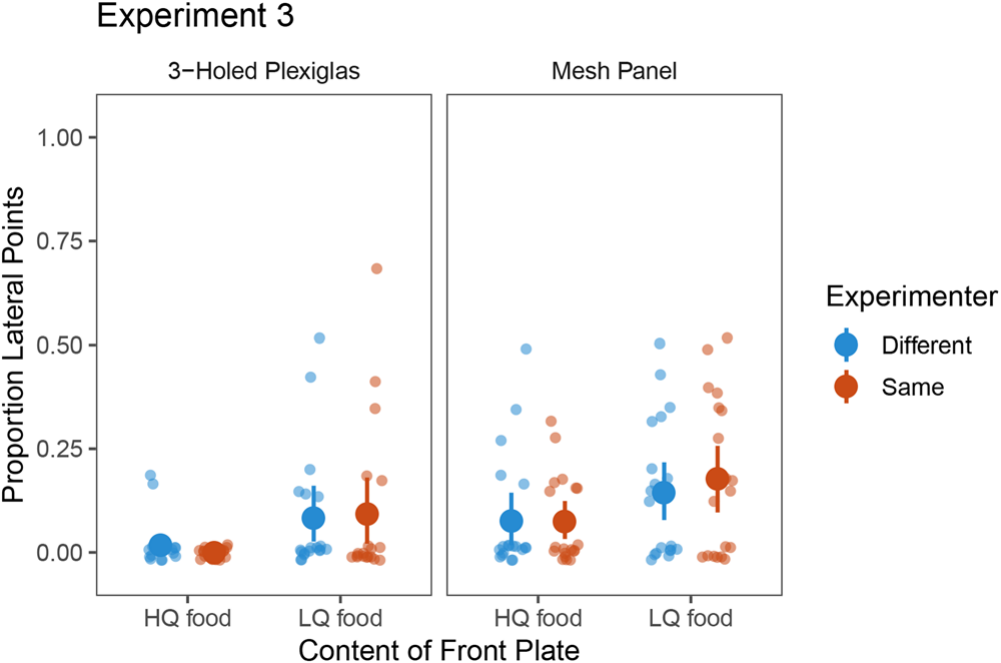
Proportion of lateral points in the four conditions of Experiment 3, when using the different panels. Larger dots indicate the means, error bars represent 95% CIs.

A GEE test revealed that Food location had a significant main effect (*χ*^2^_*(1)*_ = 12.275*, p* < 0.001), because apes produced more lateral pointing when the HQ food was in the back [*M* = 3.05 (12.71%), *SD* = 3.03] than when it was in the front [*M* = 1 (4.17%), *SD* = 1.29; see also Fig. 5]. The main effect of Panel was significant (*χ*^2^_*(1)*_ = 9.96*, p* = 0.002, for further details see the Supporting Information). The main effect of Experimenter (*χ*^2^_*(1)*_ = 1.043, *p* = 0.307) the interaction of Food location × Experimenter (*χ*^2^_*(1)*_ = 0.396, *p* = 0.529), Food location × Panel (*χ*^2^_*(1)*_ = 3.299, *p* = 0.069) and Panel × Experimenter (*χ*^2^_*(1)*_ = 3.443, *p* = 0.064) were not significant. The interaction of Food location × Experimenter × Panel could not be computed. The GLMM confirmed the results of the GEE analysis. There was a significant main effect of Food location (LRT: *χ*^2^ = 22.43, *p* < 0.001, *β* = −1.24), and Panel (LRT: *χ*^2^ = 17.29, *p* < 0.001, *β* = −1.09) and no other significant main effects or interactions. When Familiarity was included in the GLMM as a predictor we found a non-significant trend (LRT: *χ*^2^ = 3.40, *p* = 0.065, *β* = −1.11), as apes pointed slightly less often for an unfamiliar experimenter compared to a familiar one. Including Familiarity in the GLMM did not result in qualitative changes to the other predictors (for further details see the supplementary information).

### Discussion

The results of Experiment 3 were in line with the findings of Experiment 2: We found a significant main effect of Food location. Apes performed more lateral points when the HQ food item was located in the back. However, we did not find an interaction between Food location × Experimenter. This implies that regardless of the experimenter’s knowledge, apes produced more lateral points when the high quality food was further away. Therefore, the presence of an ignorant addressee who would have needed disambiguation of the indicated object did not induce an increase in the number of lateral pointing. Furthermore, the proportion of lateral pointing was again low in Experiment 3, suggesting that the additional uncertainty about the location of the desired food in the experimenter’s side did not have a facilitatory effect on the disambiguation of pointing. Taken together, the results obtained again suggest that apes adjusted their pointing to the spatial context. However, there was no evidence that non-human great apes integrate this information with the perspective of the addressee in Experiment 3. A further GLMM analysis provided similar pattern of results supporting these conclusions. Although, apes’ familiarity with experimenters might affect their willingness to produce lateral points in general, this non-significant trend did not change how they integrated spatial and social information to disambiguate the referent.

## General Discussion

As pragmatic theories of communication emphasize^3,4^, verbal and gestural communication in humans is spontaneously adjusted to the context. Here, we show that great apes also have a similar kind of flexibility in their production and modulation of pointing, nevertheless, it seems that this flexibility is restricted to the spatial layout. In line with the communicative account of gesture use, our results suggest that captive great apes not just produce pointing in an intentional way, but also spontaneously adjust this gesture to the spatial context implying that pointing is not an automatic or associated response to certain perceptual cues.

Previous studies showed that great apes use communicative gestures intentionally^11–18^ and socially, pointing more often when a human is around^24,25^, indicating that they are sensitive to the presence of humans. Furthermore, great apes keep track whether the addressee was present previously and adjust their subsequent communicative acts accordingly^38^. Importantly, however, our results in Experiment 2 and 3 provide no evidence that apes consider the particular perspective of the addressee in communicative interactions – at least in the present setup – and it remains an open question to what degree and under what circumstances they can take into account and integrate the perspective of their communicative partner.

This is in line with studies showing that apes can perform level 1 perspective taking (understanding that the other knows that an object is present; see e.g.^45^), but experience difficulties with level 2 perspective taking (understanding how the visual scene is represented by the other; see^46^). If in the present task apes would be restricted to Level 1 perspective taking the knowledgeable addressee would be represented by them as only knowing that both containers are baited, but not as knowing which container has the LQ and the HQ food. This would motivate the apes to inform the experimenter about the correct location of the HQ food item. However, the apes would also need to indicate the container in which the HQ food item is located for an ignorant addressee. This would explain why there was no difference in the amount of informative gestures evoked in the two conditions of Experiment 3.

Alternatively, great apes may be able to represent others’ perspective, however they could not integrate this information into the cognitive process, which led them to produce lateral points in order to indicate HQ food item when it was further away. Previous studies show that apes have difficulties when solving a task, which requires the integration of different kinds of information. For instance, Okamoto-Barth and Call^47^ found that apes could 1) use the presence of an object (marker) placed on top of the baited container (out of the two available) and 2) track the location of the food inside the container (which they had seen placed there) when the containers swapped locations (invisible displacement). However, there was no evidence that apes tracked the food’s invisible displacement whose presence had been solely indicated by the marker after the latter was removed and the containers swapped locations (see also^48^ for other examples of failure to integrate information from two sources). Therefore, it is possible that – instead of lack of perspective taking – apes’ behavior in Experiment 2 and 3 reflects a similar kind of inability to integrate relevant information in order to point unambiguously.

Intentional communication in non-human animals is proposed to involve goal- and recipient-directedness besides reliable congruency between the reactions of the addressee and the apparent intention of the signaler^7^. The present results indicate that, in addition to ceasing to signal or changing the signal captive great apes are also able to *modify* an existing signal as a function of spatial referential context. This flexible modification of a communicative behavior may be a further indicator of goal-directedness in communication. Moreover, the ability of great apes to adjust pointing to the situational setup may also imply that the congruency of signaler’s and recipient’s actions should be analyzed while taking the context into consideration, since depending on it, a set of distinct actions can be produced to elicit the same reaction in the addressee. For instance, as a function of the position of the pointing hole in Experiment 1 great apes produced bent pointing distally or used non-modified proximal points. Consequently, the present findings highlight the role and relevance of spatial context in intentional signaling and provides new evidence for including contextual adjustment of gestures as a further type of relevant indicator of intentional communication in non-human animals.

Overall, the present results and previous studies converge to indicate that captive great apes are able to develop at least a rudimentary capacity to communicate by using a finite set of gestural signals that they can flexibly modify or adjust to the specific spatial context in order to inform their recipient. This is especially true of their use of indexical signals, such as pointing, where successful indication and disambiguation of an intended referent inherently involves the need to adjust or modify the communicative gesture as a function of the spatial and referential context.

### Ethical approval

All subject voluntarily participated in the study and were never food deprived. Water was accessible for them ad libitum. The study was approved by the joint ethics committee of Max Planck Institute for Evolutionary Anthropology, Leipzig, and Zoo Leipzig, Germany. The present research was non-invasive and strictly adhered to the legal requirements of Germany. The methods were carried out in accordance with the relevant guidelines and regulations. Animal husbandry and research complied with the European Association of Zoos and Aquaria (EAZA) Minimum Standards for the Accommodation and Care of Animals in Zoos and Aquaria and the World Association of Zoos and Aquariums (WAZA) Ethical Guidelines for the Conduct of Research on Animals by Zoos and Aquarium.

## Data availability

The authors declare that all data supporting the findings of this study are available within the paper and in the supplementary information.

## Supplementary information

Supplementary Information

## References

[1] Grice HP (1957). Meaning. Philos. Rev..

[2] Scott-Phillips, T. C. *Speaking Our Minds: Why human communication is different, and how language evolved to make it special*. (Palgrave MacMillan, 2014).

[3] Sperber, D., & Wilson, D. *Relevance: Communication and Cognition*. (Blackwell’s, 1986).

[4] Dennett DC (1983). Intentional systems in cognitive ethology: “The Panglossian paradigm” defended. Behav. Brain Sci..

[5] Seyfarth RM, Cheney DL, Marler P (1980). Monkey responses to three different alarm calls: evidence of predator classification and semantic communication. Science.

[6] Sievers, C., Wild, M., & Gruber, T. Intentionality and Flexibility in Animal Communication in *The Routledge Handbook of Philosophy of Animal* Minds (ed. Andrews, K., Beck, J.) 333–342 (Routledge, 2017).

[7] Townsend SW (2017). Exorcising Grice’s ghost: an empirical approach to studying intentional communication in animals. Biol. Rev..

[8] Bates, E., Benigni, L., Breterthon, I., Camaioni, L., & Volterra, V. *The emergence of symbols: Cognition and communication in infancy* (Academic Press, 1979).

[9] Bates E, Camaioni L, Volterra V (1975). Performatives prior to speech. Merrill Palmer Quart.

[10] Cartmill EA, Byrne RW (2007). Orangutans modify their gestural signaling according to their audience’s comprehension. Curr. Biol..

[11] Leavens DA, Russel JL, Hopkins WD (2005). Intentionality as measured in the persistence and elaboration of communication by chimpanzees (Pan troglodytes). Child. Dev..

[12] Hostetter AB, Cantero M, Hopkins WD (2001). Differential use of vocal and gestural communication by chimpanzees (Pan troglodytes) in response to the attentional status of a human (Homo sapiens). J. Comp. Psychol..

[13] Call, J. & Tomasello, M. The gestural repertoire of chimpanzees (Pan troglodytes) in *The gestural communication of apes and monkeys* (ed. Call, J., Tomasello, M.) 17–39 (Laurence Earlbaum Associates, 2007).

[14] Pika, S. Gestures in subadult bonobos (Pan paniscus) in *The gestural communication of apes and monkeys* (ed. Call, J., Tomasello, M.) 41–67 (Laurence Earlbaum Associates, 2007).

[15] Liebal K, Pika S, Call J, Tomasello M (2004). To move or not to move: how great apes adjust to the attentional state of others. Interact. Stud..

[16] Leavens DA, Hopkins WD, Thomas RK (2004). Referential communication by chimpanzees (Pan troglodytes). J. Comp. Psychol..

[17] Hobaiter C, Byrne RW (2014). The meanings of chimpanzee gestures. Curr. Biol..

[18] Sperber D, Wilson D (2002). Pragmatics, modularity and mind-reading. Mind Lang..

[19] Wilson D, Sperber D (1994). Outline of relevance theory. Links and Letters.

[20] Liebal K, Call J (2012). The origins of non-human primates’ manual gestures. Philos. T. R. Soc. B.

[21] Byrne (2017). Great ape gestures: intentional communication with a rich set of innate signals. Anim. Cogn..

[22] Tomasello M, Carpenter M, Liszkowski U (2007). A new look at infant pointing. Child Dev..

[23] Hobaiter C, Leavens DA, Byrne RW (2014). Deictic gesturing in wild chimpanzees (Pan troglodytes)? Some possible cases. J. Comp. Psychol..

[24] Call J, Tomasello M (1994). The production and comprehension of referential pointing by orangutans (Pongo pygmaeus). J. Comp. Psychol..

[25] Leavens DA, Hopkins WD, Bard KA (1996). Indexical and referential pointing in chimpanzees (Pan troglodytes). J. Comp. Psychol..

[26] Zimmermann F, Zemke F, Call J, Gómez JC (2009). Orangutans (Pongo pygmaeus) and bonobos (Pan paniscus) point to inform a human about the location of a tool. Anim. Cogn..

[27] Kovács ÁM, Tauzin T, Téglás E, Gergely G, Csibra G (2014). Pointing as Epistemic Request: 12-month-olds Point to Receive New Information. Infancy.

[28] Liszkowski U, Carpenter M, Henning A, Striano T, Tomasello M (2004). Twelve-month-olds point to share attention and interest. Developmental Sci..

[29] Gómez JC (2007). Pointing behaviors in apes and human infants: a balanced interpretation. Child Dev..

[30] Tomasello, M. *Origins of human communication* (MIT Press, 2008).

[31] Lyn H, Greenfield PM, Savage-Rumbaugh S, Gillespie-Lynch K, Hopkins WD (2011). Nonhuman primates do declare! A comparison of declarative symbol and gesture use in two children, two bonobos, and a chimpanzee. Lang. Commun..

[32] Liszkowski U, Schäfer M, Carpenter M, Tomasello M (2009). Prelinguistic infants, but not chimpanzees, communicate about absent entities. Psychol. Sci..

[33] van der Goot MH, Tomasello M, Liszkowski U (2014). Differences in the nonverbal requests of great apes and human infants. Child Dev..

[34] Gretscher H, Tempelmann S, Haun DB, Liebal K, Kaminski J (2017). Prelinguistic human infants and great apes show different communicative strategies in a triadic request situation. PLoS ONE.

[35] Leavens DA (2015). Distal Communication by Chimpanzees (Pan troglodytes): Evidence for Common Ground?. Child Dev..

[36] Roberts AI, Vick SJ, Roberts SGB, Menzel CR (2014). Chimpanzees modify intentional gestures to coordinate a search for hidden food. Nat. Commun..

[37] Bohn M, Call J, Tomasello M (2015). Communication about absent entities in great apes and human infants. Cognition.

[38] Bohn M, Call J, Tomasello M (2016). The role of past interactions in great apes’ communication about absent entities. J. Comp. Psychol..

[39] Lyn H (2014). Apes communicate about absent and displaced objects: methodology matters. Anim. Cogn..

[40] Gonseth C, Kawakami F, Ichino E, Tomonaga M (2017). The higher the farther: distance-specific referential gestures in chimpanzees (Pan troglodytes). Biol. Letters.

[41] Hardin, J. W. & Hilbe, J. M. *Generalized Estimating Equations*. (CRC Press, 2012).

[42] Hribar A, Haun DBM, Call J (2011). Great apes’ strategies to map spatial relations. Anim. Cogn..

[43] Völter CJ, Sentís I, Call J (2016). Great apes and children infer causal relations from patterns of variation and covariation. Cognition.

[44] Kita, S. *Pointing: Where language, culture and cognition meet*. (Erlbaum, 2003).

[45] Hare B, Call J, Tomasello M (2001). Do chimpanzees know what conspecifics know?. Anim. Behav..

[46] Karg K, Schmelz M, Call J, Tomasello M (2016). Differing views: Can chimpanzees do Level 2 perspective-taking?. Anim. Cogn..

[47] Okamoto-Barth S, Call J (2008). Tracking and inferring spatial rotation by children and great apes. Dev. Psychol..

[48] Call J (2007). Apes know that hidden objects can affect the orientation of other objects. Cognition.

