# Supplementary material for: Context-sensitive adjustment of pointing in great apes
Source: Sci Rep. 2020 Jan 23;10:1048. doi: 10.1038/s41598-019-56183-7 (PMC6978377; doi:10.1038/s41598-019-56183-7)
Supplement: Supplementary file 1 — Supplementary Information [file 41598_2019_56183_MOESM1_ESM.docx]

Supplementary information

Context-sensitive adjustment of pointing in great apes

Tauzin, T., Bohn, M., Gergely, G., Call, J.

Experiment 1

Additional results

When both type of modified pointing were analyzed together, modified pointing was most frequent with the panel with the middle hole [HQ: *M* = 5.45 (45.41%), *SD* = 3.54; LQ: *M* = 0.95 (7.92%), *SD* = 1.7] compared to the sessions with the hole on the left [HQ: *M* = 2.09 (34.83%), *SD* = 1.97; LQ: *M* = 0.09 (1.5%), *SD* = 0.43] or right [HQ: *M* = 1.86 (31%), *SD* = 2.21; LQ: *M* = 0.14 (2.33%), *SD* = 0.47]. A significant difference was found in modified points when the panels with the middle vs. right holes (*χ^2^_(1)_* = 5.996*, p =* 0.014) and middle vs. left holes (*χ^2^_(1)_* = 4.195*, p <* 0.041) were compared. There was a no difference in modified points when the panels with the and left and right holes were compared (*χ^2^_(1)_* = 0.163*, p =* 0.687).

Analyzing the bent pointing at the HQ food separately (when it was not in front of a pointing hole) revealed that the average occurrence of this action was 3.91 (16.29%, *SD* = 3.64). The average of bent pointing at the LQ food (when it was not in front of a pointing hole) was 1.09 (4.54%, *SD* = 1.72). The main effect of Food location (*χ^2^_(1)_* = 18.359*, p <* 0.001) and Panel (*χ^2^_(2)_* = 8.249*, p =* 0.016) was significant according to GEE while the Food location × Panel interaction was not (*χ^2^_(2)_* = 0.119*, p =* 0.942). Subjects used more bent pointing when the pointing hole was in the middle [HQ: *M* = 2.77 (23.08%), *SD* = 2.89; LQ: *M* = 0.86 (7.17%), *SD* = 1.7] as opposed to the panels with the hole in the left [HQ: *M* = 0.55 (9.17%), *SD* = 1.26; LQ: *M* = 0.09 (1.5%), *SD* = 0.43] or right [HQ: *M* = 0.59 (9.83%), *SD* = 1.18; LQ: *M* = 0.14 (2.33%), *SD* = 0.47]. There was a significant difference between the panels with the middle vs. right holes (*χ^2^_(1)_* = 8.852*, p =* 0.003) and middle vs. left holes (*χ^2^_(1)_* = 6.312*, p =* 0.012). However, there was no significant effect when the number of bent pointing was compared in the hole on the left vs. right panels (*χ^2^_(1)_* = 0.173*, p =* 0.677).

The average number of pointing from behind the Plexiglas panel (when it was not in front of a pointing hole) at the HQ food was 5.5 (22.92%, *SD* = 5.71), while at the LQ was 0.09 (0.38%, *SD* = 0.43). Due to the fact that in two sessions there was no pointing from behind the Plexiglas an interaction could not be computed when running the full factorial GEE analysis, therefore it was removed from the model. GEE for main effects revealed there was a significant main effect of Food location (*χ^2^_(1)_* = 39.067*, p <* 0.001) and no effect of Panel (*χ^2^_(2)_* = 3.44*, p =* 0.177). There was no significant effect of Species on the total number of modified pointing (*χ^2^_(3)_* = 2.315*, p =* 0.510) according to a Kruskal-Wallis test. For further details see also Table 1.

Discussion

In Experiment 1 we found that the physical characteristics of the panels we used had a significant effect on modified pointing, mainly because bent pointing was more frequent when the panel with the middle hole was used. This might be the result of the experimental arrangement as there was no food placed proximally behind the middle hole, therefore a bent finger could not indicate unintentionally the LQ item merely due to its proximity to the food.

We also found that the results about the different type of modified pointings showed a similar pattern. Both bent pointing and pointing from behind the Plexiglas panel was more frequent when HQ food item was further away from the pointing hole suggesting that great apes not simply substituted their typical pointing with a less typical gesture, but applied different referential gestures spontaneously.

Experiment 2

Additional results

Apes performed significantly more lateral points (*χ^2^_(1)_* = 11.832*, p =* 0.001) when the mesh panel was used [LQ front: *M* = 1.43 (35.71%), *SD* = 1.71; HQ front: *M* = 0.76 (19.05%), *SD* = 1.34; No food front: *M* = 1.52 (38.1%), *SD* = 1.44] as opposed to the intact Plexiglas [LQ front: *M* = 0.24 (5.95%), *SD* = 0.89; HQ front: *M* = 0.29 (7.14%), *SD* = 0.9; No food front: *M* = 0.33 (8.33%), *SD* = 0.97], and 3-holed Plexiglas panel [LQ front: *M* = 0.48 (11.9%), *SD* = 0.93; HQ front: *M* = 0.05 (1.19%), *SD* = 0.22; No food front: *M* = 0.57 (14.29%), *SD* = 0.87]. There was no significant difference between the two type of Plexiglas panels in the number of lateral points (*χ^2^_(1)_* = 1.972*, p =* 0.160). The effect of species was not significant according to a Kruskal-Wallis test (*χ^2^_(3)_* = 3.286*, p =* 0.369). For further details see also Table 2.

Discussion

Besides the effect of Food location there was also a significant main effect of Panel and a significant interaction of Food location × Panel in Experiment 2. The main effect of Panel was due to that lateral points were the most frequent when the mesh panel was used in contrast to the Plexiglas panels, probably because the mesh provided more alternative holes to put the finger through and to indicate HQ by a lateral gesture. The significant interaction we found was presumably driven by the intact Plexiglas panel which did not differentiate between the conditions (see Fig. 4). Since an intact panel looked the same as a standard wall in the apes’ testing room, apes might consider the manipulations behind it as irrelevant for them. This might also reduce the number of all the points as well, which therefore, was lower overall.

Experiment 3

Additional results

The main effect of Panel was significant (*χ^2^_(1)_* = 9.96, *p* = 0.002) due to more lateral pointing when the mesh was used [LQ front, same experimenter: *M* = 1.05 (35.09%), *SD* = 1.08; LQ front, different experimenter: *M* = 0.95 (31.58%), *SD* = 1.08; HQ front, same experimenter: *M* = 0.47 (15.79%), *SD* = 0.7; HQ front, different experimenter: *M* = 0.42 (14.04%), *SD* = 0.84] as opposed to the Plexiglass panel [LQ front, same experimenter: *M* = 0.53 (17.54%), SD = 1.07; LQ front, different experimenter: *M* = 0.53 (17.54%), *SD* = 0.96; HQ front, same experimenter: *M* = 0.0 (0.0%), *SD* = 0.0; HQ front, different experimenter: *M* = 0.11 (3.51%), *SD* = 0.32; see also Figure 5]. The effect of Species on the number of lateral points was not significant according to a Kruskal-Wallis test (*χ^2^_(2)_* = 2.448, *p* = 0.308). For further details see also Table 3.

We investigated whether the difference in familiarity with the two different experimenter pairs had an effect on great apes’ pointing behavior. We fit a generalized linear mixed model (GLMM) with a logit link function to the data. The model was fit in *R* (version 3.6.1, R Core Team) via the function *glmer* from the package *lme4* (Bates, Maechler, Bolker & Walker, 2015). The model included Condition (same or different experimenter), Food location (HQ food item back, HQ food item front) Panel (mesh, Plexiglas), Familiarity (unfamiliar experimenters, familiar experimenters), Species (bonobo, chimpanzee, orangutan) as fixed effects and Subject as random intercept with random slopes for trial (model formula: lateral_point ~ condition + food_location + panel + familiarity + species + (trial | individual)). To compute p-values for each predictor, we compared a model including this predictor to a model lacking it using likelihood ratio tests (LRT), computed via the function *drop1*. Familiarity of experimenter did not have a strong effect on participants’ lateral pointing (LRT: *χ^2^* = 3.40, *p* = 0.065, *β* = - 1.11). Apes pointed less often for an unfamiliar experimenter compared to a familiar one. Importantly, including familiarity into the model did not change the inferences licensed by the GEE analysis reported in the main paper: There was a strong effect of Panel (LRT: *χ^2^* = 17.29, *p* < 0.001, *β* = - 1.09 – more lateral points with the mesh panel) as well as food position (LRT: *χ^2^* = 22.43, *p* < 0.001, *β* = - 1.24, more lateral points with food in the back), no effect of Condition (LRT: *χ^2^* = 0.13, *p* = 0.91) and also no difference between species (LRT: *χ^2^* = 3.09, *p* = 0.21).

Discussion

The main effect of Panel on the number of lateral points was significant in Experiment 3, since more lateral points were observed when the mesh panel was used than when the Plexiglas panel was set up. We conjecture, therefore, that similarly to Experiment 2, the mesh provided more opportunities to point from the sides or above, in contrast to the Plexiglas with which great apes had to move their hands further away if they wanted to point through a pointing hole to indicate the HQ food. The GLMM analysis revealed that there was no strong effect of familiarity with experimenter and supported the results of the GEE analyses.

| **Panel** | | Middle hole | | | | Left hole | | | | Right hole | | | | Sum | | | |
| --- | --- | --- | --- | --- | --- | --- | --- | --- | --- | --- | --- | --- | --- | --- | --- | --- | --- |
| **Blocked food** | | HQ | | LQ | | HQ | | LQ | | HQ | | LQ | | HQ | | LQ | |
| **Pointing type** | | Behind | Bent | Behind | Bent | Behind | Bent | Behind | Bent | Behind | Bent | Behind | Bent | Behind | Bent | Behind | Bent |
| **Name** | **Species** |  |  |  |  |  |  |  |  |  |  |  |  |  |  |  |  |
| Abeeku | Gorilla | 3 | 0 | 0 | 0 | 1 | 0 | 0 | 0 | 6 | 0 | 0 | 0 | 10 | 0 | 0 | 0 |
| Alex | Chimp. | 11 | 0 | 0 | 0 | 4 | 0 | 0 | 0 | 6 | 0 | 0 | 0 | 21 | 0 | 0 | 0 |
| Bambari | Chimp. | 3 | 2 | 0 | 1 | 0 | 2 | 0 | 0 | 0 | 0 | 0 | 1 | 3 | 4 | 0 | 2 |
| Bimbo | Orang. | 0 | 0 | 0 | 0 | 0 | 5 | 0 | 2 | 0 | 0 | 0 | 0 | 0 | 5 | 0 | 2 |
| Daza | Chimp. | 2 | 5 | 0 | 5 | 0 | 0 | 0 | 0 | 0 | 0 | 0 | 0 | 2 | 5 | 0 | 5 |
| Dokana | Orang. | 2 | 1 | 0 | 0 | 0 | 3 | 0 | 0 | 0 | 3 | 0 | 2 | 2 | 7 | 0 | 2 |
| Fimi | Bonobo | 1 | 3 | 2 | 0 | 3 | 0 | 0 | 0 | 2 | 3 | 0 | 0 | 6 | 6 | 2 | 0 |
| Frederike | Chimp. | 3 | 1 | 0 | 0 | 2 | 0 | 0 | 0 | 3 | 0 | 0 | 0 | 8 | 1 | 0 | 0 |
| Gemena | Bonobo | 1 | 5 | 0 | 2 | 0 | 0 | 0 | 0 | 0 | 1 | 0 | 0 | 1 | 6 | 0 | 2 |
| Hope | Chimp. | 1 | 7 | 0 | 0 | 3 | 0 | 0 | 0 | 0 | 1 | 0 | 0 | 4 | 8 | 0 | 0 |
| Jeudi | Chimp. | 2 | 1 | 0 | 0 | 0 | 0 | 0 | 0 | 0 | 0 | 0 | 0 | 2 | 1 | 0 | 0 |
| Joey | Bonobo | 5 | 6 | 0 | 0 | 1 | 0 | 0 | 0 | 5 | 0 | 0 | 0 | 11 | 6 | 0 | 0 |
| Kibara | Gorilla | 0 | 0 | 0 | 0 | 0 | 0 | 0 | 0 | 0 | 0 | 0 | 0 | 0 | 0 | 0 | 0 |
| Kumili | Gorilla | 5 | 1 | 0 | 0 | 5 | 0 | 0 | 0 | 0 | 0 | 0 | 0 | 10 | 1 | 0 | 0 |
| Lexi | Bonobo | 2 | 4 | 0 | 6 | 0 | 0 | 0 | 0 | 0 | 0 | 0 | 0 | 2 | 4 | 0 | 6 |
| Luiza | Bonobo | 0 | 5 | 0 | 1 | 0 | 0 | 0 | 0 | 0 | 0 | 0 | 0 | 0 | 5 | 0 | 1 |
| Padana | Orang. | 0 | 1 | 0 | 0 | 0 | 1 | 0 | 0 | 1 | 0 | 0 | 0 | 1 | 2 | 0 | 0 |
| Pini | Orang. | 5 | 7 | 0 | 0 | 4 | 1 | 0 | 0 | 3 | 1 | 0 | 0 | 12 | 9 | 0 | 0 |
| Raja | Orang. | 0 | 10 | 0 | 0 | 2 | 0 | 0 | 0 | 0 | 4 | 0 | 0 | 2 | 14 | 0 | 0 |
| Suaq | Orang. | 0 | 1 | 0 | 1 | 0 | 0 | 0 | 0 | 0 | 0 | 0 | 0 | 0 | 1 | 0 | 1 |
| Yasa | Bonobo | 7 | 1 | 0 | 3 | 3 | 0 | 0 | 0 | 0 | 0 | 0 | 0 | 10 | 1 | 0 | 3 |
| Yasongo | Bonobo | 6 | 0 | 0 | 0 | 6 | 0 | 0 | 0 | 2 | 0 | 0 | 0 | 14 | 0 | 0 | 0 |

Table 1. Number of modified points in the different conditions by each subject in Experiment 1. Panel refers to the Plexiglas which was used in a given session. Blocked food indicates the type of food item which was not behind the pointing hole, while pointing type differentiate between bent pointing and pointing from behind the panel

| **Panel** | | Mesh | | | Intact plexi | | | Plexi with 3 holes | | | Sum | | |
| --- | --- | --- | --- | --- | --- | --- | --- | --- | --- | --- | --- | --- | --- |
| **Food location** | | LQ front | HQ front | 0 front | LQ front | HQ front | 0 front | LQ front | HQ front | 0 front | LQ front | HQ front | 0 front |
| **Name** | **Species** |  |  |  |  |  |  |  |  |  |  |  |  |
| Abeeku | Gorilla | 4 | 4 | 3 | 0 | 0 | 0 | 0 | 0 | 1 | 4 | 4 | 4 |
| Alex | Chimp. | 4 | 1 | 4 | 4 | 4 | 4 | 2 | 0 | 2 | 10 | 5 | 10 |
| Bimbo | Orang. | 0 | 1 | 1 | 0 | 0 | 0 | 0 | 0 | 0 | 0 | 1 | 1 |
| Dokana | Orang. | 0 | 0 | 0 | 0 | 0 | 0 | 0 | 0 | 0 | 0 | 0 | 0 |
| Fimi | Bonobo | 0 | 0 | 1 | 0 | 0 | 0 | 0 | 0 | 0 | 0 | 0 | 1 |
| Frederika | Chimp. | 4 | 4 | 4 | 0 | 1 | 2 | 0 | 0 | 1 | 4 | 5 | 7 |
| Gemena | Bonobo | 0 | 2 | 1 | 0 | 0 | 0 | 0 | 0 | 0 | 0 | 2 | 1 |
| Hope | Chimp. | 1 | 0 | 1 | 0 | 0 | 0 | 0 | 0 | 0 | 1 | 0 | 1 |
| Jeudi | Chimp. | 3 | 3 | 3 | 0 | 0 | 0 | 2 | 0 | 0 | 5 | 3 | 3 |
| Joey | Bonobo | 4 | 0 | 4 | 0 | 0 | 0 | 0 | 0 | 0 | 4 | 0 | 4 |
| Kibara | Gorilla | 2 | 0 | 2 | 0 | 0 | 0 | 0 | 0 | 0 | 2 | 0 | 2 |
| Kumili | Gorilla | 0 | 0 | 0 | 0 | 0 | 0 | 2 | 0 | 1 | 2 | 0 | 1 |
| Lexi | Bonobo | 2 | 0 | 2 | 0 | 0 | 0 | 0 | 0 | 0 | 2 | 0 | 2 |
| Luiza | Bonobo | 0 | 0 | 0 | 0 | 0 | 0 | 0 | 0 | 0 | 0 | 0 | 0 |
| Padana | Orang. | 0 | 0 | 1 | 0 | 0 | 0 | 0 | 0 | 1 | 0 | 0 | 2 |
| Pini | Orang. | 0 | 0 | 0 | 0 | 0 | 0 | 0 | 0 | 1 | 0 | 0 | 1 |
| Raja | Orang. | 0 | 0 | 0 | 0 | 0 | 0 | 3 | 1 | 3 | 3 | 1 | 3 |
| Suaq | Orang. | 2 | 0 | 1 | 0 | 0 | 0 | 1 | 0 | 2 | 3 | 0 | 3 |
| Yasa | Bonobo | 4 | 0 | 3 | 0 | 1 | 0 | 0 | 0 | 0 | 4 | 1 | 3 |
| Yasongo | Bonobo | 0 | 0 | 1 | 1 | 0 | 1 | 0 | 0 | 0 | 1 | 0 | 2 |
| Zira | Chimp. | 0 | 1 | 0 | 0 | 0 | 0 | 0 | 0 | 0 | 0 | 1 | 0 |

Table 2. Number of lateral points in the different conditions by each subject in Experiment 2. Panel indicates the different sessions with a given panel. Food location specifies which food item was in front

| **Panel** | | Mesh | | | | Plexi with 3 holes | | | | Sum | | | |
| --- | --- | --- | --- | --- | --- | --- | --- | --- | --- | --- | --- | --- | --- |
| **Food location** | | LQ front | | HQ front | | LQ front | | HQ front | | LQ front | | HQ front | |
| **Experimenter(s)** | | Same | Different | Same | Different | Same | Different | Same | Different | Same | Different | Same | Different |
| **Name** | **Species** |  |  |  |  |  |  |  |  |  |  |  |  |
| Alex | Chimp. | 1 | 2 | 0 | 0 | 2 | 3 | 0 | 0 | 3 | 5 | 0 | 0 |
| Batak | Orang. | 2 | 1 | 0 | 0 | 1 | 1 | 0 | 0 | 3 | 2 | 0 | 0 |
| Dokana | Orang. | 2 | 0 | 0 | 0 | 0 | 0 | 0 | 0 | 2 | 0 | 0 | 0 |
| Fimi | Bonobo | 0 | 0 | 0 | 0 | 0 | 0 | 0 | 0 | 0 | 0 | 0 | 0 |
| Frederika | Chimp. | 1 | 0 | 1 | 3 | 0 | 1 | 0 | 0 | 1 | 1 | 1 | 3 |
| Gemena | Bonobo | 0 | 0 | 0 | 0 | 0 | 0 | 0 | 0 | 0 | 0 | 0 | 0 |
| Hope | Chimp. | 0 | 1 | 0 | 0 | 0 | 0 | 0 | 0 | 0 | 1 | 0 | 0 |
| Jeudi | Chimp. | 2 | 2 | 1 | 1 | 0 | 1 | 0 | 0 | 2 | 3 | 1 | 1 |
| Joey | Bonobo | 0 | 3 | 0 | 0 | 0 | 0 | 0 | 0 | 0 | 3 | 0 | 0 |
| Lexi | Bonobo | 0 | 2 | 1 | 0 | 0 | 0 | 0 | 0 | 0 | 2 | 1 | 0 |
| Luiza | Bonobo | 2 | 0 | 0 | 2 | 0 | 0 | 0 | 0 | 2 | 0 | 0 | 2 |
| Padana | Orang. | 3 | 2 | 0 | 0 | 4 | 3 | 0 | 0 | 7 | 5 | 0 | 0 |
| Pini | Orang. | 0 | 1 | 2 | 0 | 0 | 0 | 0 | 0 | 0 | 1 | 2 | 0 |
| Raja | Orang. | 2 | 1 | 1 | 0 | 0 | 0 | 0 | 0 | 2 | 1 | 1 | 0 |
| Suaq | Orang. | 1 | 0 | 0 | 0 | 1 | 0 | 0 | 1 | 2 | 0 | 0 | 1 |
| Tanah | Orang. | 0 | 0 | 0 | 0 | 0 | 0 | 0 | 0 | 0 | 0 | 0 | 0 |
| Yasa | Bonobo | 3 | 3 | 2 | 1 | 0 | 0 | 0 | 0 | 3 | 3 | 2 | 1 |
| Yasongo | Bonobo | 1 | 0 | 0 | 0 | 0 | 1 | 0 | 0 | 1 | 1 | 0 | 0 |
| Zira | Chimp. | 0 | 0 | 1 | 1 | 2 | 0 | 0 | 1 | 2 | 0 | 1 | 2 |

Table 3. Number of lateral points in the different conditions by the subjects in Experiment 3. Panel indicates the different sessions with a given panel. Food location specifies which food item was in front while Experimenter(s) show whether there was an experimenter change or not

References:

Bates, D., Maechler, M., Bolker, B., & Walker, S. Fitting Linear Mixed-Effects Models Using lme4. *J. Stat. Softw.* **67**, 1-48 (2015).

R Core Team. R: A language and environment for statistical computing. R Foundation for Statistical Computing, Vienna, Austria. URL http://www.R-project.org/ (2019).
